# Growth, Structure, Thermal Properties and Spectroscopic Characteristics of Nd^3+^-Doped KGdP_4_O_12_ Crystal

**DOI:** 10.1371/journal.pone.0100922

**Published:** 2014-06-26

**Authors:** Tongqing Sun, Yu Zhang, Pai Shan, Zichang Zhang, Shaolin Chen, Yongfa Kong, Jingjun Xu

**Affiliations:** 1 The MOE Key Laboratory of Weak-Light Nonlinear Photonics, Nankai University, Tianjin, People’s Republic of China; 2 School of Physics, Nankai University, Tianjin, People′s Republic of China; 3 TEDA Applied Physics Institute, Nankai University, Tianjin, People’s Republic of China; Griffith University, Australia

## Abstract

A single crystal of Nd^3+^-doped KGdP_4_O_12_ was successfully grown with the top-seeded solution growth and slow cooling (TSSG−SC) technique. It crystallizes in space group *C*2/*c* with cell parameters *a* = 7.812(2) Å, *b* = 12.307(3) Å, *c* = 10.474(2) Å, *β* = 110.84(3)° and *Z* = 4. The IR and Raman spectra also indicated that the phosphoric polyhedra of Nd:KGdP_4_O_12_ has a cyclic symmetry. The chemical composition of the crystal was analyzed and the distribution coefficient of Nd^3+^ was calculated. The crystal morphology of KGdP_4_O_12_ was identified using X-ray diffraction. The compound has good thermal stability to 920°C. Its specific heat and thermal conductivity were determined for potential applications. The spectral properties of Nd:KGdP_4_O_12_ indicates that it exhibits broad absorption and emission bands, which are attributed to low symmetry of the crystal. The broad absorption band around 798 nm has a full-width at half-maximum (FWHM) of 14.8 nm and is suitable for AlGaAs laser diode pumping. Moreover, 5 at% Nd^3+^-doped KGdP_4_O_12_ crystal has a long luminescence lifetime of 300 μs and a high quantum efficiency of 96%.

## Introduction

With the development of the diode-pumped solid-state (DPSS) lasers based on neodymium-doped crystals, research on new laser host materials has gained much interest [1]. Nd:YAG crystal is still the most common Nd^3+^ laser material because of its good physical and laser properties, but it is limited to low Nd^3+^-doped concentration and narrow absorption bandwidth near the AlGaAs diode emission wavelength of 808 nm. Because the emission wavelength of the laser diode increases at 0.2−0.3 nm·K^−1^ of the laser device, the temperature stability of the laser diode needs to be tightly controlled. Therefore, it is necessary to explore new and more efficient crystals for DPSS lasers. Furthermore, the absorption band of laser crystals close to the laser output of AlGaAs (*λ* = 808 nm) needs to have a large full-width at half-maximum (FWHM).

Recently, some Nd^3+^-doped laser crystals with broad absorption bands have been reported. They include host crystals with disordered structures such as NaLn(WO_4_)_2_ (Ln = Y or Gd, *I*4_1_/*a*) [2], [3], SrLaGa_3_O_7_ (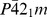) [4], Ca_3_La_2_(BO_3_)_4_ (*Pnma*) [5], Li_3_Ba_2_Ln_3_(MO_4_)_8_ (Ln = Y, La or Gd, M = W or Mo, *C*2/*c*) [6−11], KBaGd(MO_4_)_3_ (M = W or Mo, *C*2/*c*) [12], [13] and CaNb_2_O_6_ (*Pbcn*) [14]. These species can induce inhomogeneous broadening of optical spectra from a disordered crystal lattice. Mixed crystals such as garnet (Lu_x_Y_1−x_)_3_Al_5_O_12_ [15] and rare earth oxyorthosilicate Ln_2_SiO_5_ (*C*2/*c*) (LuYSiO_5_, GdYSiO_5_ and LuGdSiO_5_) [16−18] can enhance the structure disorder. Many of the host crystals above have low symmetry, especially the monoclinic system *C*2/*c* space group. Due to the strong anisotropy of their physical properties and biaxiality, host crystals with low symmetry may enrich spectroscopic properties of doped active ions [19].

The title compound, KGdP_4_O_12_, belongs to the broader family of condensed phosphates including those double phosphates of alkali and lanthanide ions with the general formula M^I^Ln^III^(PO_3_)_4_. The structure of M^I^Ln^III^(PO_3_)_4_ is highly dependent not only on the sizes of the alkali and the lanthanide ions but also on the crystallization environment. Double tetra-metaphosphates of potassium and gadolinium have low symmetry (monoclinic system) including three structural types with different space groups: *P*2_1_, *P*2_1_/*n* and *C*2/*c*. The first two types are written as KGd(PO_3_)_4_ in general and are double polyphosphates. Here, the phosphoric anions have a long-chain geometry of 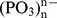. The *C*2/*c* is a cyclotetraphosphate, and these phosphoric anions have a cyclic geometry of [P_4_O_12_]^4−^; hence its formula is usually written as KGdP_4_O_12_. The growth and structure of KGd(PO_3_)_4_ (*P*2_1_) crystal was reported by Parreu et al [20], [21] and it is isostructural to KNd(PO_3_)_4_ [22]–a famous Nd stoichiometric laser material. The KGd(PO_3_)_4_ (*P*2_1_) can be used as a self-frequency doubling (SFD) laser host material because of its noncentrosymmetrical structure and has attracted more attention [23−26]. KGdP_4_O_12_ crystallized in space group *C*2/*c* and KGd(PO_3_)_4_ in *P*2_1_/*n* were successively synthesized in phosphoric acid by Naïli et al [27], [28].

Compared to other double phosphates of alkali and lanthanide ions with the general formula M^I^Ln^III^(PO_3_)_4_, KGdP_4_O_12_ has the following characteristics as a new laser host material. First, Gd^3+^ is much closer to Nd^3+^ in size; hence KGdP_4_O_12_ is easily substituted with Nd^3+^. Second, raw materials for preparing KGdP_4_O_12_ are easily available and very cheap in comparison with double metaphosphates of cesium or rubidium. Third, the spectroscopic spectrum of doped active ions may be broadened because of its low symmetry in the space group *C*2/*c*. Finally, the KGdP_4_O_12_ crystal has a cyclic geometry that is difficult to cleave in contrast with compounds having long chain geometry.

To the best of our knowledge, the crystal growth of KGdP_4_O_12_ has not yet been reported. Here, we have grown a single crystal of Nd^3+^-doped KGdP_4_O_12_ with high quality and determined its structural character to be a cyclic geometry via single crystal structural analysis and IR and Raman spectroscopy. The spectral characteristics of the Nd:KGdP_4_O_12_ crystal were studied through its absorption spectrum, emission and excitation fluorescent spectra, and fluorescence decay curve. Finally, we measured its thermal stability, specific heat and thermal conductivity.

## Materials and Methods

### 1. Crystal growth

The crystal growth used a single-zone vertical tubular electric furnace with a Kantal AF heater. The temperature of the furnace was measured near the heater with a B-type thermocouple and was regulated with a SHIMADEN FP21 type temperature controller.

We grew Nd:KGdP_4_O_12_ crystals with the top-seeded solution growth and slow cooling (TSSG−SC) technique from a self-flux. The starting material was prepared by mixing Nd_2_O_3_ (4N), Gd_2_O_3_ (4N), K_2_CO_3_ (99.0%) and NH_4_H_2_PO_4_ (99.0%) at a molar ratio of 0.05∶0.95∶3∶12 in an agate mortar. This was then transferred into a Φ60×60 mm^2^ Pt crucible to melt in batches. During the melting, a stoichiometric Nd_2_O_3_, Gd_2_O_3_, K_2_CO_3_ and NH_4_H_2_PO_4_ generated the title compound KGd_0.95_Nd_0.05_P_4_O_12_. An excess of K_2_CO_3_ and NH_4_H_2_PO_4_ reacted to produce potassium metaphosphate compound, which served as a self-flux to avoid foreign ions during the crystal growth. The typical chemical reactions are shown below.
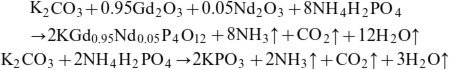



There was marked bubbling of NH_3_, CO_2_ and H_2_O, and the mixture was heated slowly to a melt. The melt was homogenized at 900°C with a stirrer until the melt was clear and there were no bubbles. Meanwhile, the crucible was rotated alternately clockwise and counterclockwise at 15 revolutions per minute for complete homogenization. The oscillating crucible rotation was also necessary during the crystal growth for effective mass transport. The coldest position in the solution was 0.5 cm below the melt surface and the temperature gradient in the axial direction was 1.3°C·cm^−1^. After several attempts, the melt temperature decreased to 812°C. Meanwhile, a thick wire of platinum attached to a corundum rod was introduced into the furnace and was placed above the melt. About 10 min later, the platinum wire was immersed below the melt surface as a seed. While maintaining the oscillating crucible rotation, the melt temperature was reduced at 0.4°C·d^−1^ until it reached 794°C. During this process, the solute first crystallized on the platinum wire, which served as the seed for larger crystals.

**Figure 1:**
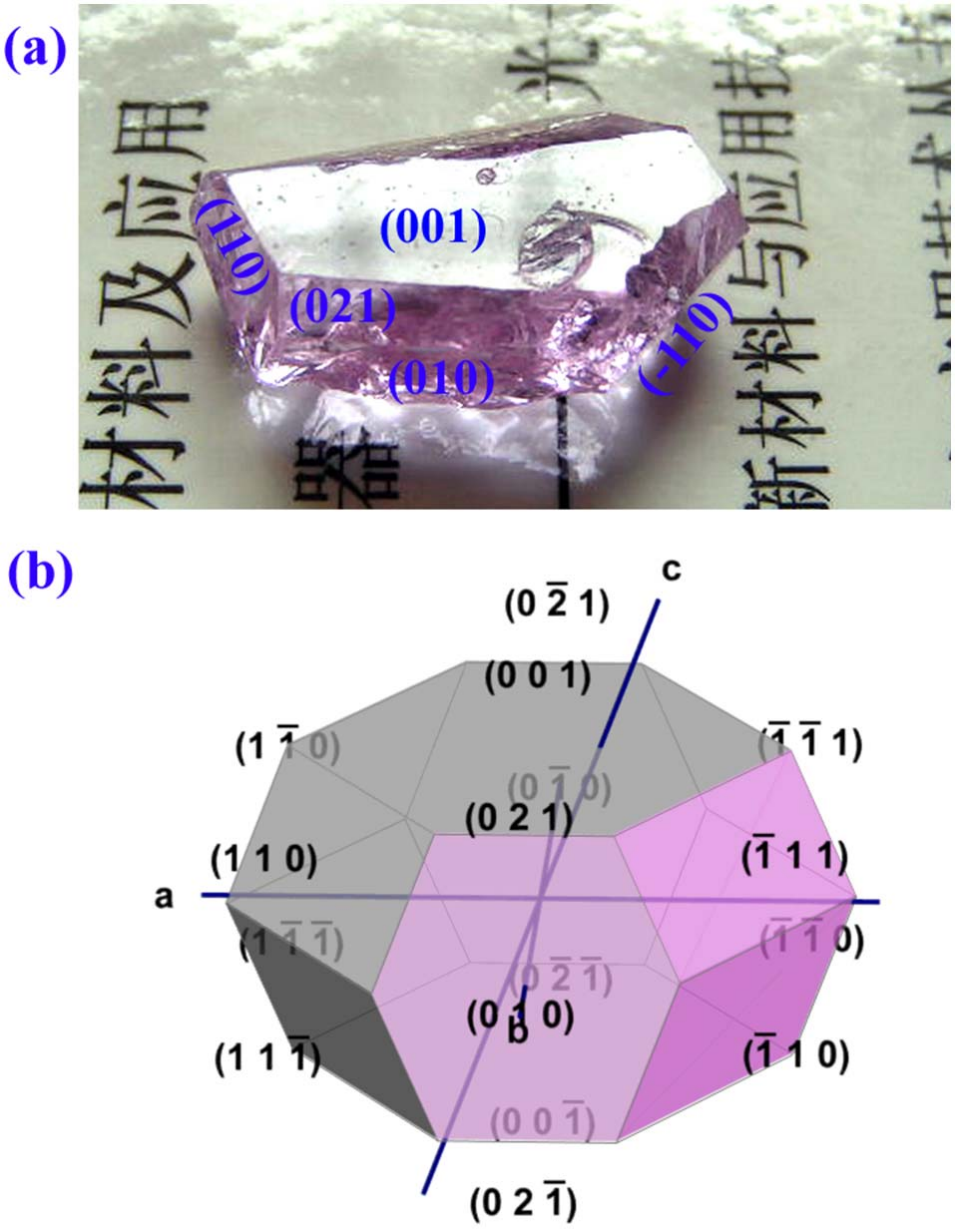
Photograph (a) of the as-grown single crystal with a size of 20 mm×10 mm×5 mm and the theoretical morphology (b) of the Nd:KGdP_4_O_12_ crystal.

After the growth was completed, the crystals were removed from the solution. The furnace was cooled to room temperature slowly and then some single crystals clustering around the platinum wire were taken out of the furnace. Figure 1(a) shows that the biggest one of these had good transparency with no cracks or inclusions. The colour of the crystal shows that the neodymium ion had already entered the crystal lattice. The stoichiometry of the crystal was analyzed by inductively coupled plasma−atomic emission spectrometer (ICP−AES) (Thermo IRIS Advantage). The density of the Nd:KGdP_4_O_12_ crystal at room temperature (23°C) was measured using a density unit of Sartorius balance (YDK01−C) by the buoyancy method.

### 2. X-ray diffraction analysis

The X-ray diffraction (XRD) technique was used to analyze several aspects of the as-grown Nd:KGdP_4_O_12_ single crystal such as determining its structure, identifying the crystalline phase, studying the crystal morphology as well as crystallinity.

A single crystal of Nd:KGdP_4_O_12_ cut from the as-grown crystal was used for indexing and intensity data collections on a Rigaku Saturn CCD area detector diffractometer using Mo *K*α graphite monochromated radiation (*λ* = 0.71073 Å). The structure was resolved with the SHELXS-97 computer utility, and refined by a full-matrix least-squares method with SHELXL-97 software.

Polycrystalline powder of Nd:KGdP_4_O_12_ was prepared from the as-grown single crystal. The powder X-ray diffraction (PXRD) pattern of Nd:KGdP_4_O_12_ was recorded at room temperature with a Fangyuan DX2700 (Dandong, China) powder diffractometer using graphite monochromatized Cu *K*α radiation in the 2*θ* range 10°−70° with a step of 0.02°.

An XRD goniometer was used to identify and orientate the crystalline forms that comprised the morphological habit for the as-grown single crystal had well-developed facets. XRD was also used to check the quality of the as-grown Nd:KGdP_4_O_12_ crystal. The crystal in Figure 1(a) was polished and used as a sample. The rocking curve of the (004) diffraction crystal face was obtained on an XRD diffractometer (PANalytical X’Pert PRO) equipped with a four-crystal Ge (022) monochromator. The setting of the X-ray tube (PW3373/10) was 40 kV and 10 mA. The step time and step size were 0.1 s and 0.001°, respectively.

### 3. IR and Raman spectroscopy

The molecular spectroscopy and lattice vibrations of Nd:KGdP_4_O_12_ were studied with Fourier transform infrared (FTIR) and Raman spectroscopy. The IR spectrum of a KBr pressed pellet of the powered sample was recorded from 4000 to 400 cm^−1^ on a Nicolet Magna-IR 560 ESP FTIR spectrometer. The as-grown crystal shown in Figure 1(a) was also studied on a Renishaw inVia Raman microscope. The output wavelength of the excited CW argon laser was 514 nm.

### 4. Thermal analysis

The thermal stability of Nd:KGdP_4_O_12_ was measured with both thermogravimetry and differential scanning calorimetry (TG−DSC) using a Netzsch STA 449C from 40 to 1000°C at 10°C·min^−1^. The specific heat of the Nd:KGdP_4_O_12_ crystal was also measured by DSC using a simultaneous thermal analyzer (Netzsch DSC 200 F3) in an atmosphere of N_2_.

The thermal diffusivity was measured by the laser flash method using a laser flash apparatus (Netzsch LFA 457 Nanoflash) in the 25–300°C range at intervals of 50°C. A 0.95 mm×4 mm×4 mm sample was used to carry out the measurements whose {001} faces were polished and coated with graphite on both sides.

### 5. Spectroscopic characterization

A 4.30-mm thick sample was polished and used to measure the spectroscopic properties. The polished facet was {001}. The unpolarized absorption spectrum was measured on a Hitachi UV−Vis−NIR spectrophotometer (U4100) from 175 to 2000 nm. The fluorescence spectra were recorded using an Edinburgh Instruments FLS920 spectrophotometer with a xenon lamp as the excitation source. The incident light and fluorescence were dispersed with two M300 monochromators using ruling gratings from Bentham Instruments. A cooled Hamamatsu R5509-72 photomultiplier was used for detection. The decay curve was measured using a pulsed xenon lamp as the pump source. The pulse duration was 10 ns, and the excitation wavelength was 808 nm. All the measurements were performed at room temperature (300 K). The splitting of the Nd^3+^ emission band was also studied at 10 K using the same FLS920 spectrophotometer equipped with a close cycle helium cryostat (Advanced Research Systems DE202).

## Results and Discussion

### 1. Crystal growth and chemical composition

As mentioned above, KGdP_4_O_12_ is a polymorphous compound. In addition to the *C*2/*c* phase, it has two isomers, i.e. KGd(PO_3_)_4_ in *P*2_1_ and *P*2_1_/*n*. Both KGdP_4_O_12_ in *C*2/*c* and KGd(PO_3_)_4_ in *P*2_1_/*n* can be prepared by crystallizing KH_2_PO_4_ and Gd_2_O_3_ from phosphoric acid below 550°C [27], [28]. Because of the lower crystallization temperature and the resulting higher viscosity, it is difficult to grow bulk crystals of Nd:KGdP_4_O_12_. Parreu grew a KGd(PO_3_)_4_ crystal in *P*2_1_ from a melt made from NH_4_H_2_PO_4_, K_2_CO_3_ and Gd_2_O_3_ at a higher temperature [20]. In studying the crystallization region of KGd(PO_3_)_4_ (*P*2_1_) in the ternary system Gd_2_O_3_−K_2_O−P_2_O_5_, six neighbouring phases were found, and KGdP_4_O_12_ in *C*2/*c* was one of them. By choosing the ratio in the melt composition, we successfully grew bulk crystals of Nd:KGdP_4_O_12_ (Figure 1(a)). The composition of a melt influences the formation of phase. A similar situation occurred to a BaTeMo_2_O_9_ polymorphous compound recently reported–the α and β crystal phases can be grown from a melt with different compositions [29].

The chemical composition of the as-grown crystal was analyzed by ICP−AES (Table 1). The chemical formula of the crystal corresponds to KGd_0.95_Nd_0.05_P_4_O_12_, which is consistent with results of single crystal diffraction. The distribution coefficient of the neodymium ion at the structural sites of gadolinium ion is defined as *K*_Nd_ = ([Nd]/([Nd]+[Gd]))_crystal_/([Nd]/([Nd]+[Gd]))_solution_. This can be calculated from the data in Table 1 and the stoichiometry of the starting materials. The value (0.984) is very close to 1 because the ionic radius of Nd^3+^ is only slightly bigger than that of Gd^3+^. This means that the active Nd^3+^ ion can be easily doped in the KGdP_4_O_12_ host crystal, and that the composition inside a single crystal would be homogeneous in general.

**Table 1:** The result of chemical analysis for the as-grown crystal of 5 at% Nd^3+^-doped KGdP_4_O_12_.

Element (mass%)	K (%)	Gd (%)	Nd (%)	P (%)
Calculated	7.64	29.20	1.41	24.22
Experimental	7.27	28.98	1.38	23.86

### 2. Crystal structure

Nd:KGdP_4_O_12_ crystallizes in the monoclinic space group *C*2/*c* with cell parameters *a* = 7.812(2) Å, *b* = 12.307(3) Å, *c* = 10.474(2) Å, *β* = 110.84(3)° and *Z* = 4. Information regarding crystal data, data collection and refinement is given in Table S1. The atomic coordinate information is given in Table S2, and the anisotropic displacement parameter is given in Table S3. The lengths of P−O, Gd(Nd)−O and K−O bonds are listed in Table S4, and the bond valences have been calculated according to the Brown and Altermatt parameters [30] for the structure of Nd:KGdP_4_O_12_. The bond valence sums are reasonable for both cations and anions. The crystal structure of Nd:KGdP_4_O_12_ we show here is consistent with that of undoped KGdP_4_O_12_ reported by Ettis et al [27]. The Nd^3+^-active ion occupies the lattice site of Gd^3+^. The density of a single crystal of Nd:KGdP_4_O_12_ at 23°C is 3.54 g·cm^−3^. This agrees well with the calculated value from the crystallographic data.

**Figure 2:**
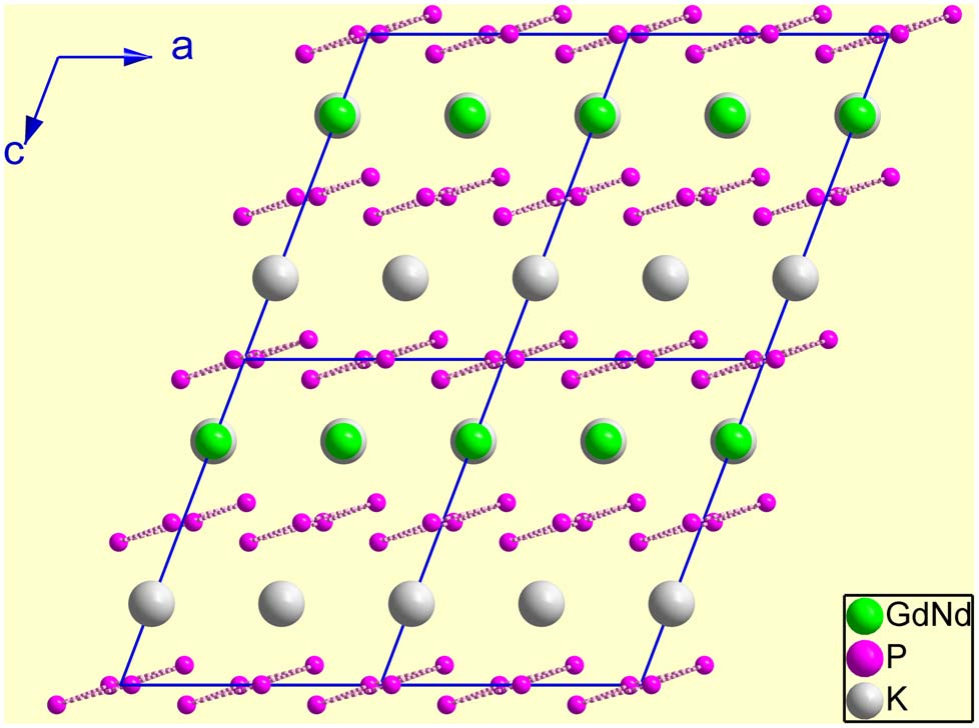
Structure projection of Nd:KGdP_4_O_12_ along the [010] direction. (Here, all the oxygen atoms have been omitted.).

The basic structural unit of Nd:KGdP_4_O_12_ is a centrosymmetric cyclotetraphosphate ring anion of [P_4_O_12_]^4−^ that is composed of four PO_4_ tetrahedra. Each PO_4_ tetrahedron shares its two corners (i.e. bridging oxygen) with the others. The four phosphorus atoms of each [P_4_O_12_]^4−^ ring lie in the same plane due to symmetry, and all the oxygen atoms are either above or below the P_4_ plane. As shown in projection into the *ac* plane (Figure 2), the [P_4_O_12_]^4−^ rings form layers parallel to (001) at *z* = 0 and 1/2. A previous description [27] suggesting that these layers were perpendicular to the [001] direction is erroneous because the normal of the (001) faces is different from the [001] direction. We note that the [P_4_O_12_]^4−^ rings stack in the structure similar to fish scales. The P_4_ plane, i.e. (0.2908, −0.0415, 0.9559), is not parallel to (001), and the interfacial angle between them is 17.08(2)°.

The Gd(Nd)O_8_ polyhedra and KO_10_ polyhedra link the [P_4_O_12_]^4−^ rings to form a three-dimensional framework. The eight vertexes of the Gd(Nd)O_8_ polyhedra come from terminal oxygens of the PO_4_ polyhedra, and they belong to six [P_4_O_12_]^4−^ rings that are equal in the two adjacent layers. The Gd(Nd)O_8_ polyhedra do not share any oxygen atoms and are isolated by the PO_4_ and KO_10_ polyhedra. Ettis et al [27] erroneously reported the shortest distance between the two rare earth ions to be 5.269 Å. Thus, they thought that it was small compared to the other alkali metal and rare earth tetraphosphates. Actually, the shortest Gd(Nd)−Gd(Nd) distance in the Nd:KGdP_4_O_12_ crystal is 6.0059 Å, which is actually relatively long for tetraphosphates.

Figure S1 shows the experimental XRD pattern of the pulverized Nd:KGdP_4_O_12_ crystal as well as our simulated pattern. The peak positions and diffraction intensities are consistent between the experimental and simulated XRD patterns. This confirms that our proposed structure is accurate.

For a laser crystal, the mechanical strength is due to the host material. Cleavage behaviour of the host crystal is related not only to the bond strength but also the geometry. There are three kinds of chemical bonds in potassium gadolinium phosphates: P−O, Gd−O and K−O. According to their covalent character, P−O bonds are much stronger than Gd−O and K−O bonds. The K−O bonds are the weakest of all. This is confirmed by the calculated results of bond valences shown in Table S5. The KGd(PO_3_)_4_ structures (both *P*2_1_ and *P*2_1_/*n*) are formed by intrachain P−O bonds and interchain Gd−O and K−O bonds. The P−O bonds run along the [100] direction for KGd(PO_3_)_4_ in *P*2_1_ and [101] for KGd(PO_3_)_4_ in *P*2_1_/*n* to generate long chains, respectively. Therefore, the KGd(PO_3_)_4_ crystals have the strongest strength along the above [100] or [101] directions and cleavage very easily occurs between the chains. In KGdP_4_O_12_ however, the GdO_8_ polyhedra and KO_10_ polyhedra link [P_4_O_12_]^4−^ rings to form a three-dimensional framework, and the strengths along the different directions are quite similar. Therefore, the Nd:KGdP_4_O_12_ crystal does not have a prominent cleavage behaviour. During the processing of Nd:KGdP_4_O_12_ crystals, no crack and cleavage as seen in KGd(PO_3_)_4_ crystals was found.

### 3. Crystal morphology and rocking curve

The as-grown crystal has some well-developed facets (Figure 1(a)) oriented using an XRD goniometer. The habit contains crystalline forms {001}, {010}, {110} and {021}. The measured interfacial angles among the facets were in good agreement with the calculated angles.

The morphology of a crystal reflects its structure. Taking into account only the point group and the crystal cell parameters, we established the theoretical morphological scheme of the Nd:KGdP_4_O_12_ crystal via the WinXMorph software [31] using the Bravais-Friedel and Donnay-Harker (BFDH) law. This morphological scheme is shown in Figure 1(b). Table 2 lists the crystalline forms {hkl}, equivalent faces and corresponding *d*_hkl_ for Nd:KGdP_4_O_12_.

**Table 2:** The crystalline forms {hkl} observed in the Nd:KGdP_4_O_12_ crystal and the corresponding *d*_hkl_ arranged by decreasing sense.

{hkl}	*d*_hkl_ (Å)
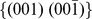	9.7688
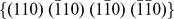	6.2790
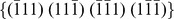	6.2204
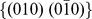	6.1535
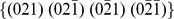	5.2096
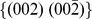	4.8944

While the as-grown crystal did not show a completely ideal morphology due to the restriction of the crystal growth method, all the predicted crystalline forms can be found on the as-grown single crystal. It should be noted that the 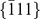 facet was smaller and limited by the growth conditions. Thus, it was quite pristine and we did not identify 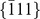 for the as-grown crystal. The narrow tetragonal facet (021) on the as-grown crystal shows that {021} has a faster growth rate, which is consistent with the predicted scheme. The ideal morphology of KGdP_4_O_12_ is like a drum and hexagonal {001} facets reveal much more than other facets similar to drumheads. This relates to the structural feature of KGdP_4_O_12_ in which [P_4_O_12_]^4−^ rings regularly array to form layers parallel to (001). The distance between the two [P_4_O_12_]^4−^ rings adjacent to the two layers is larger than that from the same layer.

The rocking curve of the (004) diffraction plane of the as-grown Nd:KGdP_4_O_12_ crystal is presented in Figure S2. The diffraction peak is intense and with good symmetry without splitting, and its FWHM value is 0.006°. These results demonstrate that the as-grown Nd:KGdP_4_O_12_ crystal has a nearly perfect lattice structure.

### 4. IR and Raman spectra

**Figure 3:**
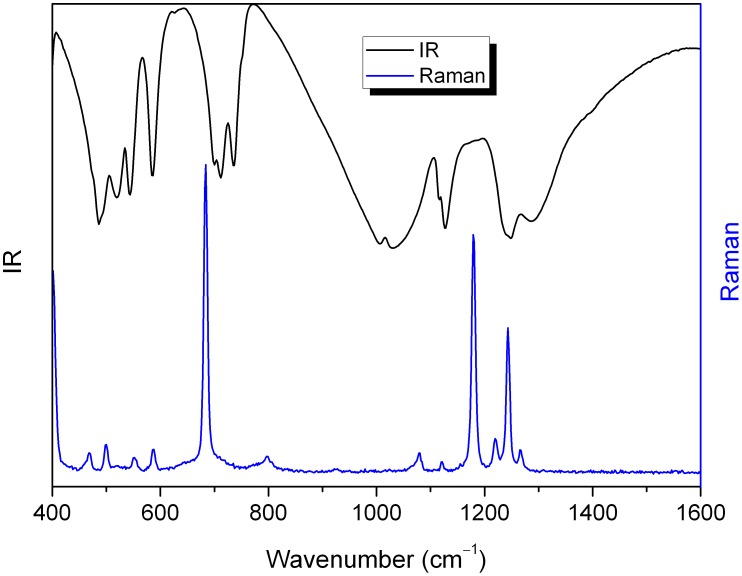
The IR and Raman spectra of Nd:KGdP_4_O_12_ at room temperature.

The IR and Raman spectra of Nd:KGdP_4_O_12_ at room temperature are shown in Figure 3. The vibration frequencies above 600 cm^−1^ and their corresponding assignment are listed in Table S5 and are based on published findings [27], [32]. In the low-frequency region below 600 cm^−1^, it is very difficult to distinguish the symmetric and antisymmetric bending modes of the O−P−O and P−O−P groups.

Compared to other cyclotetraphosphate compounds, we notice an absence of bands in the 750−1000 cm^−1^ region of the IR spectrum. This result is in agreement with the cyclic structure of Nd:KGdP_4_O_12_. The non-coincidence of the observed bands in the IR and Raman spectra indicates that the [P_4_O_12_]^4−^ anion is centrosymmetric, which is in agreement with the above structural results.

A distinguishing characteristic also exits in the Raman spectrum. The symmetric stretching vibration of the P−O−P linkage–*ν*_s_(P−O−P)–has a single peak at 684 cm^−1^. This is the strongest of all the Raman vibration peaks. However, for *ν*_s_(P−O−P) of KGd(PO_3_)_4_ (*P*2_1_), two peaks are observed in the range of 660–730 cm^−1^ and have the same intensity [24]. These are less than one half of the intensity of the *ν*_s_(O−P−O). That is because of the symmetrical difference in the crystal structures and the different positions of the lanthanide and alkali ions. The obvious differences between the IR and Raman spectra of KGdP_4_O_12_ and KGd(PO_3_)_4_ highlight how IR and Raman spectroscopy can identify the structure of alkali-metal lanthanide metaphosphates.

### 5. Thermal properties

Generally, double phosphate compounds are not stable at high temperatures. To measure the decomposition, a TG−DSC analysis for the Nd:KGdP_4_O_12_ crystal was performed. The TG−DSC curves of the Nd:KGdP_4_O_12_ crystal are given in Figure S3. A single sharp endothermic peak is observed at 920°C, which exhibits the characteristics of a first-order phase transition. The sample weight of Nd:KGdP_4_O_12_ does not show representative variation in the measurement temperature up to 1000°C. Apart from a crystalline phase, an amorphous phase was formed in the sample cell after the DSC measurement. The M^I^Ln^III^P_4_O_12_ compounds often decompose irreversibly into lanthanide tri-metaphosphates and alkali metal metaphosphates [33]. We thus conclude that the exothermic peak at 920°C may be related to a decomposition of Nd:KGdP_4_O_12_ in accordance with the reaction:
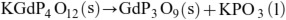
The decomposition temperature of the Nd:KGdP_4_O_12_ crystal is much higher than that of KGd(PO_3_)_4_ (*P*2_1_) (878°C) [20]. The difference in stability between these two potassium gadolinium metaphosphates can be explained by comparing their crystal data. The lengths of P−O bonds vary from 1.4752 to 1.6009 Å for KGdP_4_O_12_. However, the P−O bond lengths vary from 1.4414 to 1.6886 Å for KGd(PO_3_)_4_. That is, the PO_4_ tetrahedra in KGd(PO_3_)_4_ are heavily distorted as are the GdO_8_ polyhedra. The Gd(Nd)−O distances in the Nd:KGdP_4_O_12_ are from 2.3665 to 2.4189 Å with an average of 2.3933 Å. The Gd−O distances in KGd(PO_3_)_4_ vary widely from 2.2903 to 2.4767 Å with an average of 2.4059 Å. In addition, the volume per formula of KGdP_4_O_12_ (235.3 Å^3^) is smaller than that of KGd(PO_3_)_4_ (240.4 Å^3^). Thus, the thermal stability of the cyclic *C*2/*c* space group should be better than that of the chain structure in the *P*2_1_ space group.

For laser crystal materials, the damage threshold and possible laser applications can be greatly influenced by the specific heat. The specific heat is also an important value used to calculate thermal conductivity. Figure S4 shows that the constant pressure specific heat of the Nd:KGdP_4_O_12_ crystal varies as a function of the temperature. The specific heat of the Nd:KGdP_4_O_12_ crystal at room temperature (25°C) is 0.521 J·g^−1^·K^−1^. This is comparable to Nd:YAG (0.59 J·g^−1^·K^−1^) and Nd:YVO_4_ (0.51 J·g^−1^·K^−1^) [34]. Because Nd:YAG and Nd:YVO_4_ both have a high optical damage threshold, it follows that Nd:KGdP_4_O_12_ should also have a high damage threshold [5]. The specific heat increases almost linearly from 0.485 to 0.799 J·g^−1^·K^−1^ with temperature increases from −10 to 510°C. This suggests that the Nd:KGdP_4_O_12_ crystal can tolerate even more thermal energy at a high temperature.

**Figure 4:**
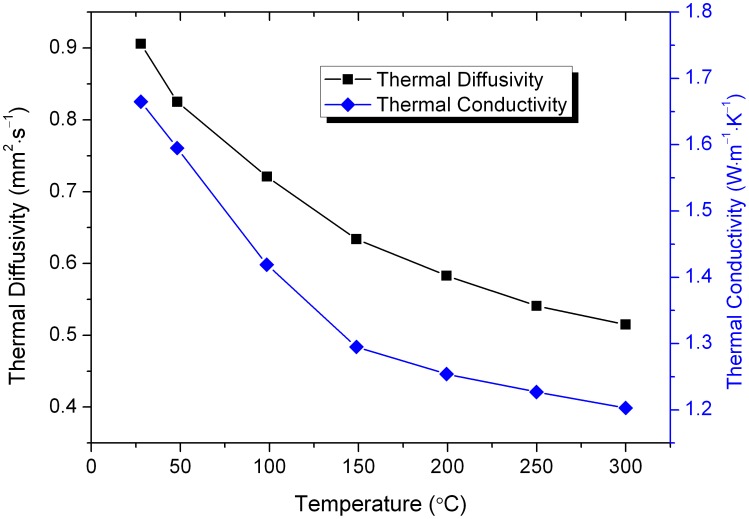
Thermal diffusivities and thermal conductivities of the Nd:KGdP_4_O_12_ crystal along the c* direction.

Figure 4 shows the thermal diffusivity and thermal conductivity of the Nd:KGdP_4_O_12_ crystal. The thermal conductivity (*κ*) was calculated using the measured thermal diffusivity and specific heat according to *κ* = *λρC*_p_, where *λ*, *ρ* and *C*_p_ denote the thermal diffusivity, density and specific heat of the crystal at the same temperature, respectively. The thermal diffusivity of the crystal is 0.906 mm^2^⋅s^−1^ along the direction perpendicular to (001), i.e. the c* direction, at 27.8°C. The calculated thermal conductivity of the crystal is 1.66 W⋅m^−1^⋅K^−1^ along the c* direction correspondingly. This value is similar to that of Nd:SrLaGa_3_O_7_ [4] and larger than those of some laser crystals with broad absorption such as NaLn(WO_4_)_2_ [2], [3] and Ca_3_La_2_(BO_3_)_4_ [5]. In laser designs, the thermal loading causes a temperature gradient in the crystal and leads to thermal expansion that results in thermal lensing and other thermo-optic effects. All these effects would cause a decline in the quality of the laser beams and even crack the active crystal. The deposited heat would be easily transferred to the environment if the laser crystal possesses high thermal conductivity–this would minimize the thermal loading effects.

It is clearly shown in Figure 4 that the thermal diffusivity and thermal conductivity component of the Nd:KGdP_4_O_12_ crystal decreases with increasing temperature. Thermal conductivity is dominated primarily by its phonon thermal conductivity for the dielectric. This has two important determinants: the heat capacity and the phonon mean free path. Though the heat capacity of the Nd:KGdP_4_O_12_ crystal increases with temperature, the phonon-phonon scattering becomes much stronger and the phonon mean free path markedly decreases. Therefore, the final result is that the thermal conductivity decreases with temperature increases.

### 6. Absorption spectrum

**Figure 5:**
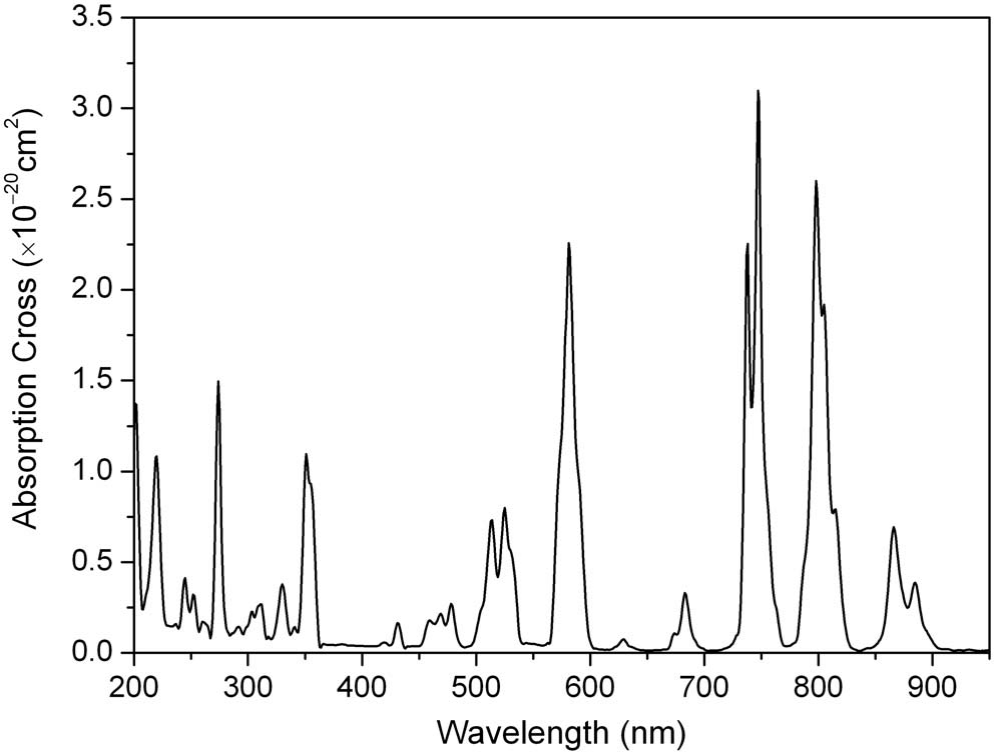
Absorption spectrum of the Nd:KGdP_4_O_12_ crystal at room temperature.

Figure 5 shows the unpolarized absorption spectrum of the Nd:KGdP_4_O_12_ crystal at room temperature. The UV cut off is below 200 nm. This means that a large band gap would increase the damage resistivity of the crystal. All absorption lines are due to the 4*f* ^3^–4*f* ^3^ transition of the Nd^3+^ ions. Very strong absorption lines occur near 581, 747 and 798 nm, corresponding to the transitions of ^4^ *I*_9/2_→^2^ *G*_7/2_+^4^ *G*_5/2_, ^4^ *I*_9/2_→^4^ *S*_3/2_+^4^ *F*_7/2_ and ^4^ *I*_9/2_→^2^ *H*_9/2_+^4^ *F*_5/2_, respectively. The interesting feature of this spectrum relative to the potential application in the diode-pumped laser, is the strong absorption band with a FWHM of 14.8 nm near 798 nm. This is close to the laser output of the AlGaAs laser diode. It is well known that temperature control of the laser diode is crucial, because the emission wavelength increases with temperature. Large line-width in the Nd:KGdP_4_O_12_ crystal is very suitable for laser diode pumping because it is not temperature dependent.

The Judd-Ofelt theory was used to calculate the optical parameters of the electric dipole transition within the 4*f* electronic configuration of the Nd^3+^ ion. Important parameters included the oscillator intensity parameters (Ω_λ_), spontaneous emission probability (*A*), fluorescence branching ratio (*β*) and radiative lifetime (*τ*_r_) [35], [36]. A detailed calculation procedure was used similar to literature [37]. Nine absorption bands were used to obtain the intensity parameters (Ω_2,4,6_), which are Ω_2_ = 1.79×10^–20 ^cm^2^, Ω_4_ = 3.48×10^–20 ^cm^2^ and Ω_6_ = 6.05×10^–20 ^cm^2^, respectively. The values of the experimental (*f*_exp_) and calculated (*f*_cal_) oscillator strengths are listed with the absorption cross sections (*σ*) in Table 3. The root mean square error (*δ*) between *f*_exp_ and *f*_cal_ is 6.97×10^−8^. The properties of ^4^ *F*_3/2_→^4^ *I_J_*_’_ (*J*’ = 9/2, 11/2, 13/2, 15/2) transitions are well known. These are the main channels of a Nd^3+^ laser and depend only on the values of Ω_4_ and Ω_6_ because the reduced matrix elements |<||*U* ^(2)^||>| for these transitions are zero. The spectroscopic quality parameter (*X* = Ω_4_/Ω_6_) is 0.57, which is less than 1 and indicates that the emission to the ^4^ *I*_11/2_ manifold is more feasible than that to ^4^ *I*_9/2_ manifold. This result can be confirmed by the fluorescence emission spectrum (see below). In this regard, it is similar to the most common laser materials based on the Nd^3+^ ion including Nd:YVO_4_ and Nd:YAG. Table 4 presents the spontaneous emission probability (*A*), radiative branching ratio (*β*) and radiative lifetime (*τ*_r_) of the multiplet ^4^ *F*_3/2_. It can be seen that the calculated branching ratios are in good agreement with the experimental ones obtained from the measured fluorescence spectrum later.

**Table 3:** The barycenter wavelength, experimental and calculated oscillator strengths, and absorption cross section of the Nd:KGdP_4_O_12_ crystal at room temperature.

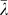 (nm)	^4^ *I*_9/2_→*J*’ manifold	*f*_exp_ (×10^−6^)	*f*_cal_ (×10^−6^)	*σ* (×10^−20 ^cm^2^)
874	^4^ *F*_3/2_	2.04	2.04	0.68
802	^4^ *F*_5/2_+^2^ *H*_9/2_	7.86	7.82	2.59
745	^4^ *F*_7/2_+^4^ *S*_3/2_	8.75	8.79	3.09
683	^4^ *F*_9/2_	0.80	0.66	0.32
581	^4^ *G*_5/2_+^2^ *G*_7/2_	11.46	11.46	2.25
520	^2^ *K*_13/2_+^4^ *G*_7/2_+^4^ *G*_9/2_	4.98	5.00	0.79
470	^2^ *K*_15/2_+^2^ *G*_9/2_+^2^ *D*_3/2_+^4^ *G*_11/2_	1.34	1.26	0.26
431	^2^ *P*_1/2_	0.45	0.47	0.15
353	^4^ *D*_3/2_+^4^ *D*_5/2_+^2^ *I*_11/2_+^4^ *D*_1/2_	9.49	9.52	1.09

**Table 4:** The calculated spontaneous emission probability, radiative branching ratios, radiative lifetime and experimental branching ratios of ^4^ *F*_3/2_ multiplet of Nd^3+^ in the Nd:KGdP_4_O_12_ crystal.

Transition	^4^ *F*_3/2_→^4^ *I*_9/2_	^4^ *F*_3/2_→^4^ *I*_11/2_	^4^ *F*_3/2_→^4^ *I*_13/2_	^4^ *F*_3/2_→^4^ *I*_15/2_
*A* (s^−1^)	1112	1705	366	17
*β*_cal_ (%)	34.8	53.3	11.4	0.5
*β*_exp_ (%)	28.6	58.8	12.6	−
*τ*_r_ (×10^−6 ^s)	312			

### 7. Fluorescence spectra analysis

**Figure 6:**
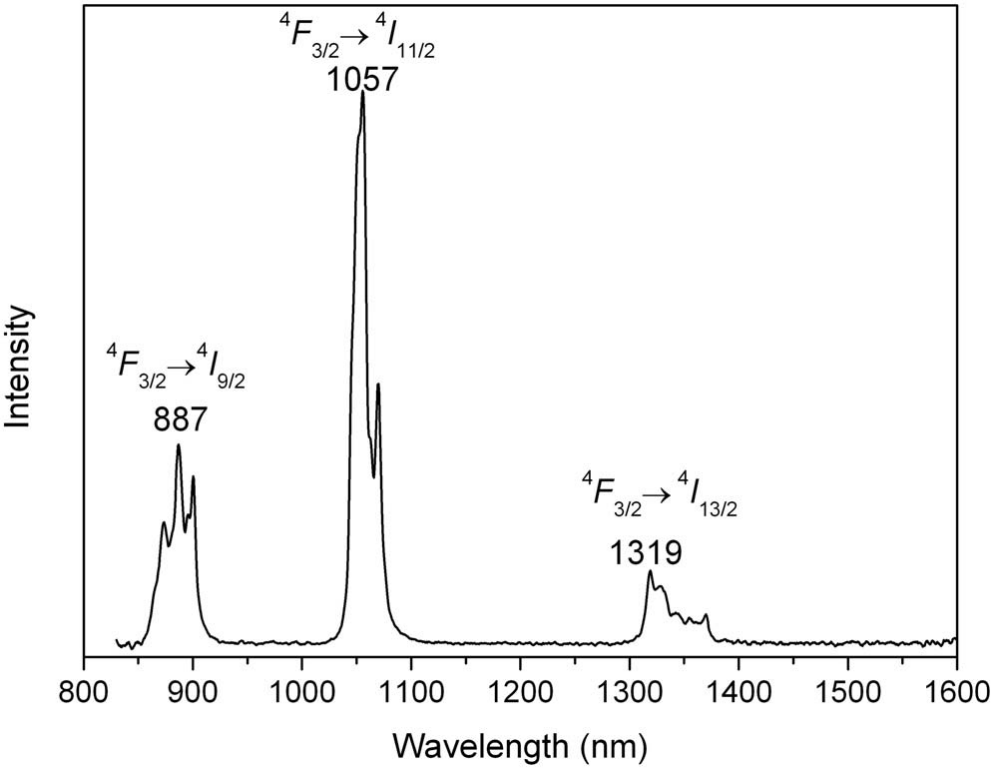
Emission fluorescence spectrum of the Nd:KGdP_4_O_12_ crystal at room temperature.

The room temperature fluorescence spectrum of the Nd:KGdP_4_O_12_ crystal at 808 nm excitation was recorded from 830 to 1600 nm (Figure 6). Three emission bands corresponding to the ^4^ *F*_3/2_ →^4^ *I*_9/2_, ^4^ *I*_11/2_ and ^4^ *I*_13/2_ transitions are observed at 860–920 nm, 1040–1080 nm and 1300–1380 nm, respectively. The room temperature emission FWHM of the transition ^4^ *F*_3/2_→^4^ *I*_11/2_ is 14 nm. This is evidence of the dominant contribution of inhomogeneous broadening to the spectra line-width in the low symmetry structure. The emission cross sections for the laser channel from ^4^ *F*_3/2_ to ^4^ *I*_9/2_, ^4^ *I*_11/2_ and ^4^ *I*_13/2_ were calculated through the Füchtbauer-Ladenburg (FL) formula [12], [18]. These values are 1.11×10^−20^, 6.25×10^−20^ and 2.13×10^−20 ^cm^2^, respectively.

**Figure 7:**
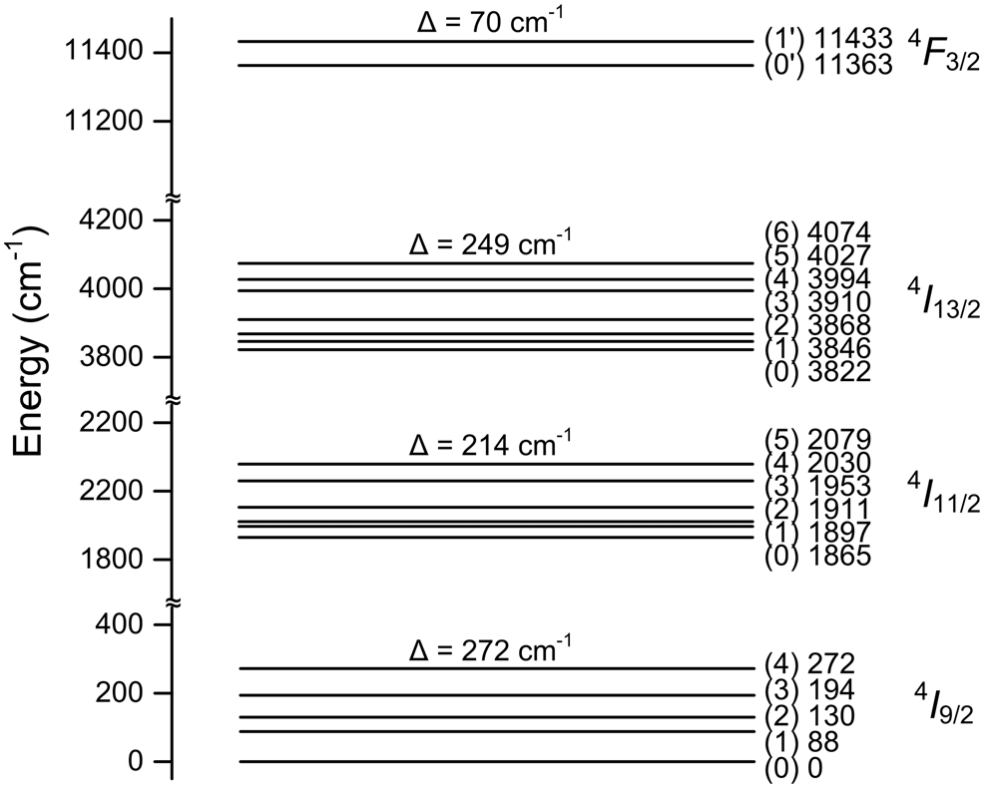
Stark structure of the energy levels of the Nd^3+^ ion in the KGdP_4_O_12_ crystal.

The emission spectra of the Nd:KGdP_4_O_12_ at low temperature were studied to determine the Stark sublevels of the ^4^ *I*_13/2_ and ^4^ *I*_11/2_ excited multiplets and the ground state multiplet ^4^ *I*_9/2_. Figure S5 shows the emission spectra of Nd:KGdP_4_O_12_ at 10K and 300K. These correspond to the transitions from ^4^ *F*_3/2_ to ^4^ *I*_9/2_, ^4^ *I*_11/2_ and ^4^ *I*_13/2_ levels, respectively. Figure 7 shows a schematic diagram of the Stark sublevels of Nd^3+^ in KGdP_4_O_12_ crystals obtained from the low temperature emission spectra. The ^4^ *F*_3/2_ term is split into two components with *Δ* = 70 cm^−1^. The low value of the crystal field splitting indicates that the crystal field of the KGdP_4_O_12_ host is weak. This is due to weak interactions between rare earth ions and oxygen atoms in contrast with the strong covalent bonds between P and O. In this regard, the Nd:KGdP_4_O_12_ crystal is similar to other double phosphates of alkali and lanthanide ions [38], [39].

The fluorescence lifetime (*τ*_f_) of the ^4^ *F*_3/2_ energy level of Nd^3+^ in the Nd:KGdP_4_O_12_ crystal was determined to be 300 µs by fitting the decay curve exponentially (Figure S6). The long fluorescence lifetime would be beneficial to high energy storage during laser operation. Though the interatomic distance (6.0059 Å) between the nearest rare earth ions for KGdP_4_O_12_ is smaller than that for KGd(PO_3_)_4_ (*P*2_1_) (6.5865 Å), it does not obviously affect the fluorescence lifetime. This is a function of the isolated GdO_8_ polyhedra in KGdP_4_O_12_. The *τ*_f_ of the Nd:KGd(PO_3_)_4_ crystal (*P*2_1_) is 246 µs with a very low doping concentration (0.6 at%) [38]. The values of Nd:LaP_5_O_14_ and Nd:GdP_5_O_14_ crystals are around 310 µs when doped with ∼1 at% Nd^3+^ [40].

It is well known that the *τ*_f_ of Nd^3+^ ions generally decreases with increasing Nd^3+^ concentrations due to the interactions between the Nd^3+^ ions, i.e. the concentration quenching effect. However, the Nd:KGdP_4_O_12_ crystal still has a long fluorescence lifetime of 300 µs under a higher doping concentration of 5 at%. Versus the above phosphates, the KGdP_4_O_12_ crystal is a distinct laser host material with a very low concentration quenching for Nd^3+^ because the Nd:KGdP_4_O_12_ crystal ensures a long fluorescence lifetime even under a high active ion concentration.

The calculated *τ*_r_ of the ^4^ *F*_3/2_ energy level is 312 µs (Table 4). Thus, the fluorescent quantum efficiency (*η* = *τ*_f/_ *τ*_r_) of the ^4^ *F*_3/2_ level is 96%. For solid-state laser materials, low crystal field strength leads to weak electron-phonon interactions and further leads to high quantum efficiency. The previous data describing weak Stark splitting of the Nd^3+^ energy levels further supports the calculated result showing that Nd:KGdP_4_O_12_ has a high quantum efficiency.

Some spectroscopic properties of the Nd:KGdP_4_O_12_ crystal are listed in Table 5 as well as those of other Nd^3+^-doped crystals including YAG and YVO_4_ crystals with ordered structures and SrLaGa_3_O_7_ and KBaGd(MoO_4_)_3_ crystals with disordered structures. The FWHM value of Nd:KGdP_4_O_12_ at *λ*_a_ is not only much larger than those of Nd:YAG and Nd:YVO_4_ crystals, but also larger than those of Nd:SrLaGa_3_O_7_ and Nd:KBaGd(MoO_4_)_3_ crystals. This suggests that the Nd:KGdP_4_O_12_ crystal can be pumped more effectively by AlGaAs laser diode, but not be restricted to the temperature stability of the output wavelength in the laser diode. The quality factor (*M*) of a laser material is proportional to its doping concentration (*N*) and fluorescence lifetime (*τ*_f_). A high value of *M* generally means a low oscillation threshold in subsequent laser operations. Therefore, the high doping concentration and long fluorescence lifetime of Nd:KGdP_4_O_12_ makes it possible to achieve a continuous wave laser action. In addition, Nd:KGdP_4_O_12_ can be used as a tunable laser material with a very broad emission band near 1060 nm.

**Table 5:** Comparison of spectroscopic properties of the Nd:KGdP_4_O_12_ crystal with other Nd^3+^-doped crystals.

Crystal	Nd:KGdP_4_O_12_	Nd:SrLaGa_3_O_7_	Nd:KBaGd(MoO_4_)_3_	Nd:YVO_4_	Nd:YAG
Nd^3+^ (at%)	5.0	1.0	0.91	1.0	1.0
Crystal system	Monoclinic	Tetragonal	Monoclinic	Tetragonal	Cubic
Growth method	TSSG	Czochralski	TSSG	Czochralski	Czochralski
Peak absorption wavelength*λ*_a_ (nm)	798	808	804	808.7	808
FWHM at *λ*_a_ (nm)	14.8	8	9	2	0.7
Peak emission wavelength*λ*_e_ (nm)	1057	1061	1069	1064	1064
FWHM at *λ*_e_ (nm)	14	14	24	1.1	0.8
Fluorescence lifetime (µs)	300	310	141	84	230
Ref.	This work	[4]	[12]	[41]	[34]

## Conclusions

We successfully grew a macro-defect-free single crystal of 5 at% Nd^3+^-doped KGdP_4_O_12_ with very good crystallinity using TSSG−SC from self-flux. To the best of our knowledge, both the growth of the bulk crystals and doping with neodymium are shown here for the first time in KGdP_4_O_12_. The Nd:KGdP_4_O_12_ crystallizes in space group *C*2/*c*, and the phosphoric anions have a cyclic geometry of [P_4_O_12_]^4−^. The crystal has good chemical stability and does not show cleavage like its isomer KGd(PO_3_)_4_. The distribution coefficient of neodymium ion is very close to 1, so gadolinium ion can be evenly substituted. The absorption and fluorescence spectra of the Nd:KGdP_4_O_12_ crystal were investigated at room temperature. The peak absorption cross section at 798 nm is 2.59×10^−20 ^cm^2^ with a FWHM of 14.8 nm. Such a broad FWHM in the absorption band is suitable for InGaAs laser diode pumping. The radiation and fluorescence lifetimes of the excited state ^4^ *F*_3/2_ are 312 and 300 μs, respectively. These result in a high luminescent quantum efficiency of 96%. The emission from the ^4^ *F*_3/2_ energy level to the ^4^ *I*_11/2_ manifold is more feasible than that of the ^4^ *I*_9/2_ manifold. The peak emission cross section at 1057 nm (corresponding to ^4^ *F*_3/2_→^4^ *I*_11/2_) is 6.25×10^−20 ^cm^2^. In addition, the KGdP_4_O_12_ crystal presents positive thermal characteristics. It has a good thermal stability with decomposition at 920°C. At room temperature, the specific heat and the thermal conductivity along the c* direction are 0.521 J·g^−1^·K^−1^ and 1.66 W⋅m^−1^⋅K^−1^, respectively. In summary, the Nd:KGdP_4_O_12_ crystal may be regarded as a potential solid-state laser material for laser diode pumping.

## Supporting Information

Figure S1 **The experimental XRD pattern of Nd:KGdP_4_O_12_ from polycrystalline powder and the simulated pattern from single crystal data.**
(DOCX)

Figure S2 **XRD rocking curve of the (004) diffraction plane of the as-grown Nd:KGdP_4_O_12_ single crystal.**
(DOCX)

Figure S3 **TG and DSC curves of the Nd:KGdP_4_O_12_ crystal.**
(DOCX)

Figure S4 **Specific heat versus temperature curve of the Nd:KGdP_4_O_12_ crystal.**
(DOCX)

Figure S5 **Emission fluorescence spectra of the Nd:KGdP_4_O_12_ crystal at 10K and 300K.**
(DOCX)

Figure S6 **Fluorescence decay curve of the ^4^** ***F*** **_3/2_ manifold of the Nd:KGdP_4_O_12_ crystal at room temperature.**
(DOCX)

Table S1 **Crystal data, data collection and refinement of Nd:KGdP_4_O_12_.**
(DOCX)

Table S2 **Atomic coordinates and equivalent isotropic displacement parameters of Nd:KGdP_4_O_12._**
(DOCX)

Table S3 **Anisotropic displacement parameters (Å^2^) of Nd:KGdP_4_O_12_.**
(DOCX)

Table S4 **Bond lengths and bond valences in the Nd:KGdP_4_O_12_ crystal.**
(DOCX)

Table S5 **Frequencies (cm^−1^) and assignments of IR absorption and Raman scattering for Nd:KGdP_4_O_12_.**
(DOCX)
