# Supplementary material for: Growth, Structure, Thermal Properties and Spectroscopic Characteristics of Nd3+-Doped KGdP4O12 Crystal
Source: PLoS One. 2014 Jun 26;9(6):e100922. doi: 10.1371/journal.pone.0100922 (PMC4072700; doi:10.1371/journal.pone.0100922)

**Figure S1.** The experimental XRD pattern of Nd:KGdP_4_O_12_ from polycrystalline powder and the simulated pattern from single crystal data.


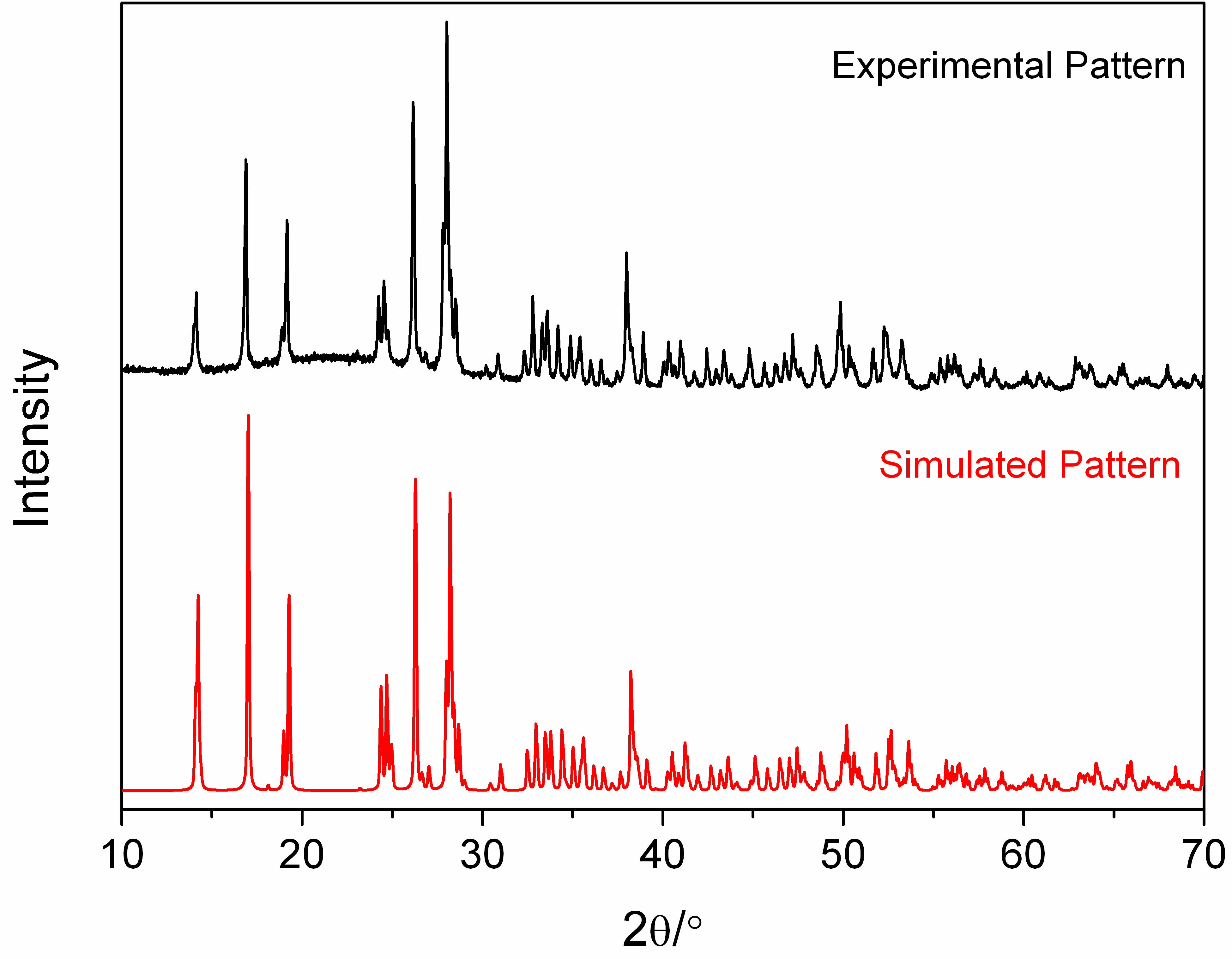

Supplement: Figure S1 — The experimental XRD pattern of Nd:KGdP4O12 from polycrystalline powder and the simulated pattern from single crystal data. (DOCX) [file pone.0100922.s001.docx]
