# Supplementary figures and images for: Growth, Structure, Thermal Properties and Spectroscopic Characteristics of Nd3+-Doped KGdP4O12 Crystal
Source: PLoS One. 2014 Jun 26;9(6):e100922. doi: 10.1371/journal.pone.0100922 (PMC4072700; doi:10.1371/journal.pone.0100922)

**Figure S2.** XRD rocking curve of the (004) diffraction plane of the as-grown Nd:KGdP4O12 single crystal.


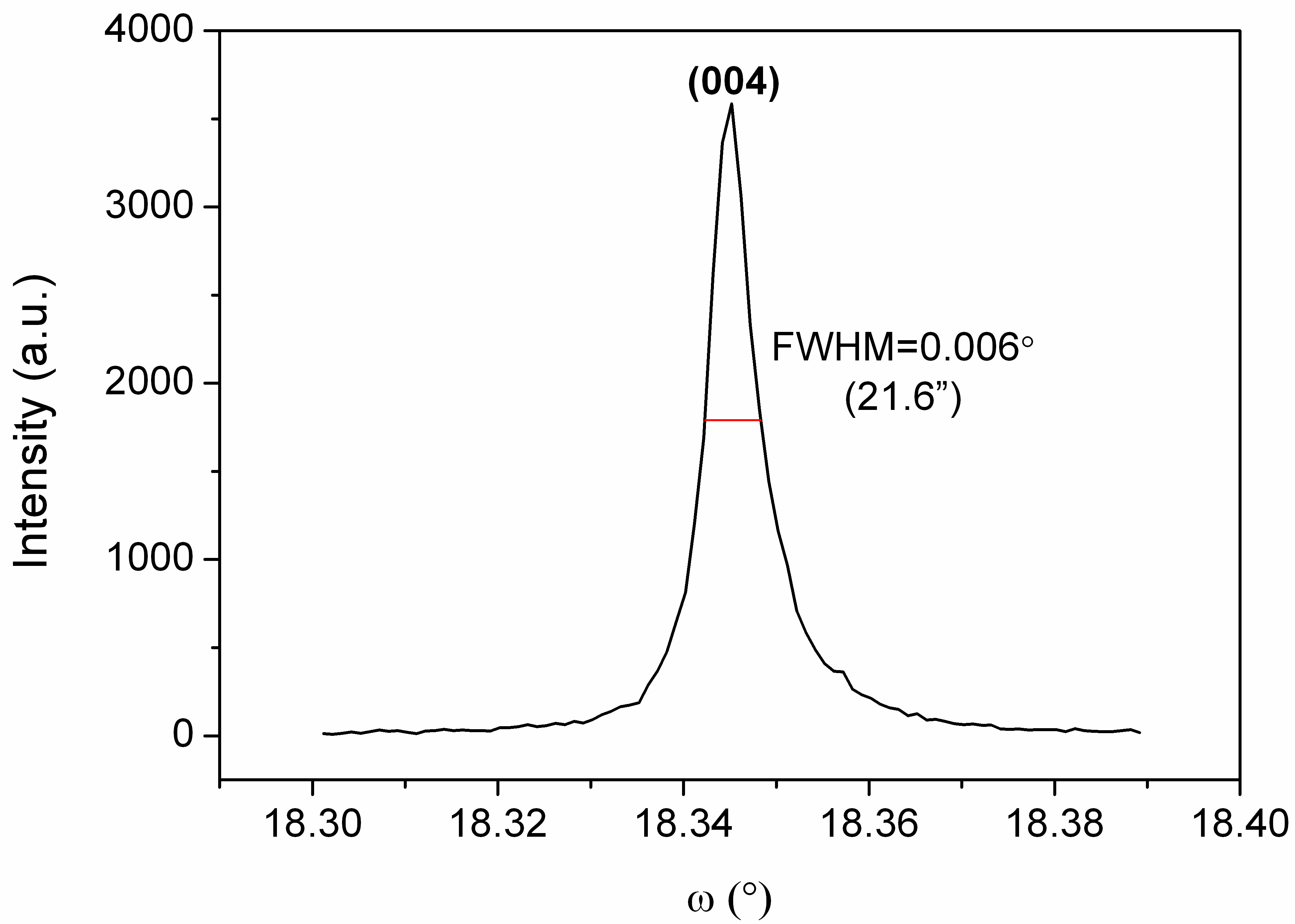

Supplement: Figure S2 — XRD rocking curve of the (004) diffraction plane of the as-grown Nd:KGdP4O12 single crystal. (DOCX) [file pone.0100922.s002.docx]

**Figure S3.** TG and DSC curves of the Nd:KGdP4O12 crystal.


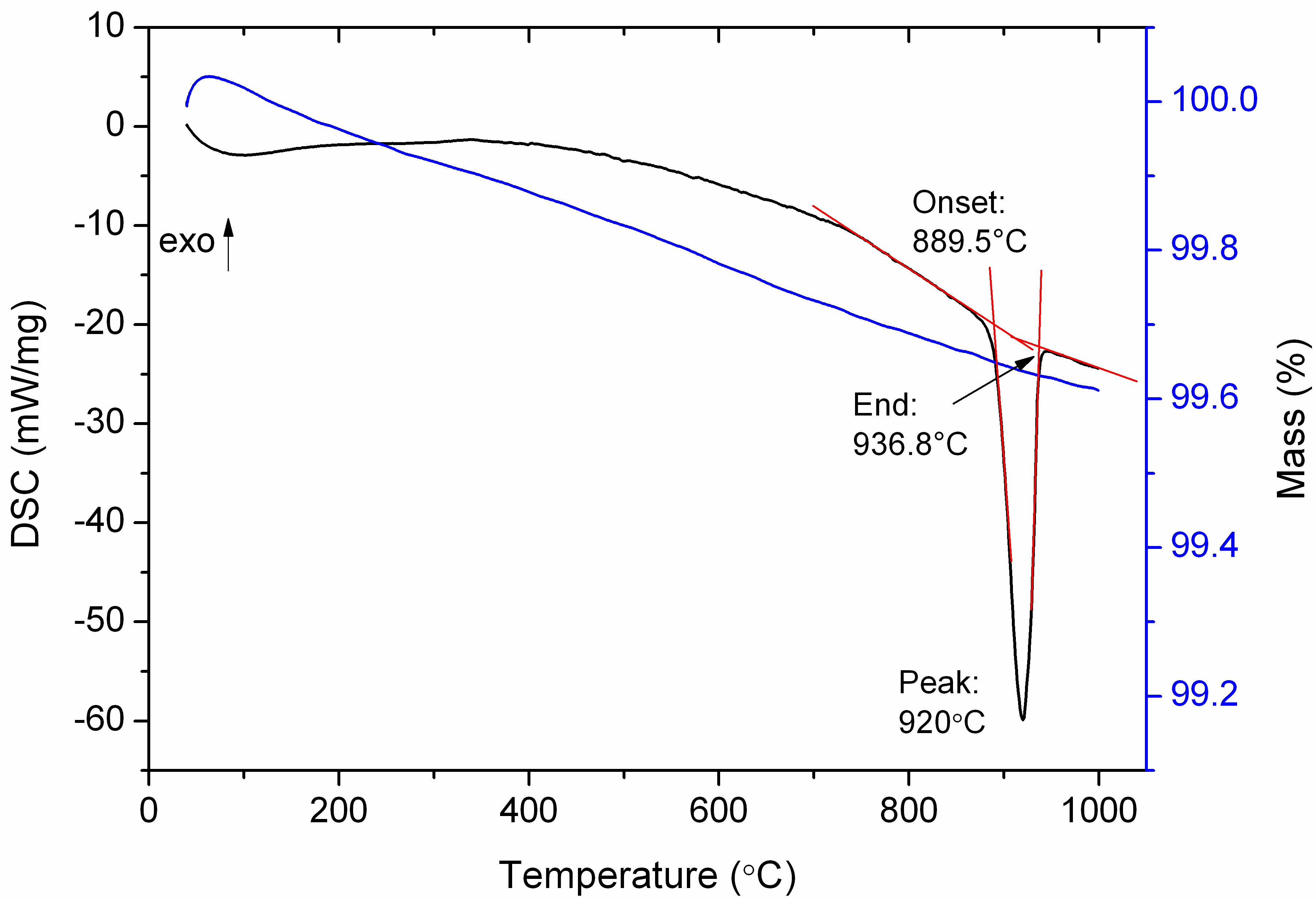

Supplement: Figure S3 — TG and DSC curves of the Nd:KGdP4O12 crystal. (DOCX) [file pone.0100922.s003.docx]

**Figure S4.** Specific heat versus temperature curve of the Nd:KGdP4O12 crystal.


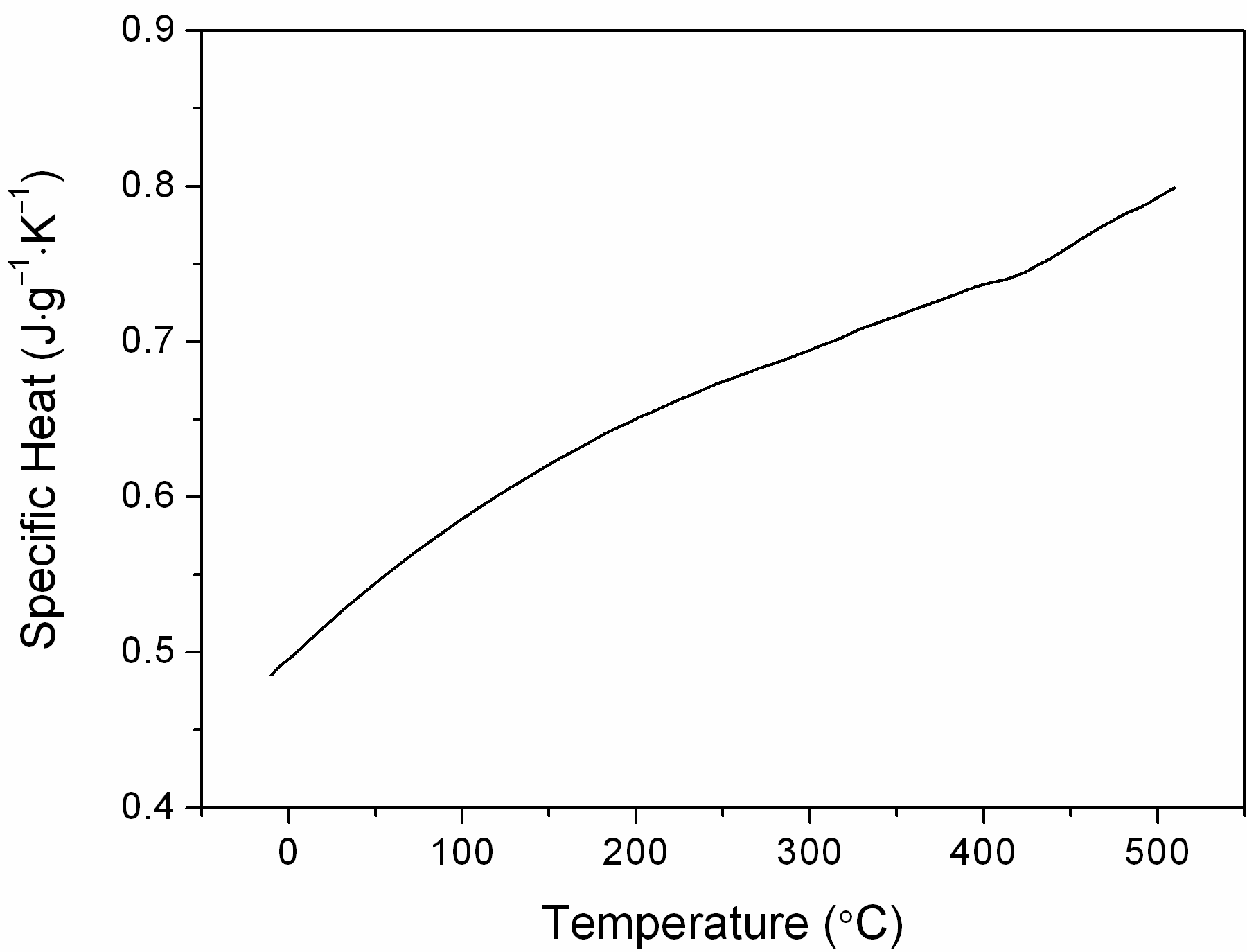

Supplement: Figure S4 — Specific heat versus temperature curve of the Nd:KGdP4O12 crystal. (DOCX) [file pone.0100922.s004.docx]

**Figure S5.** Emission fluorescence spectra of the Nd:KGdP4O12 crystal at 10K and 300K.


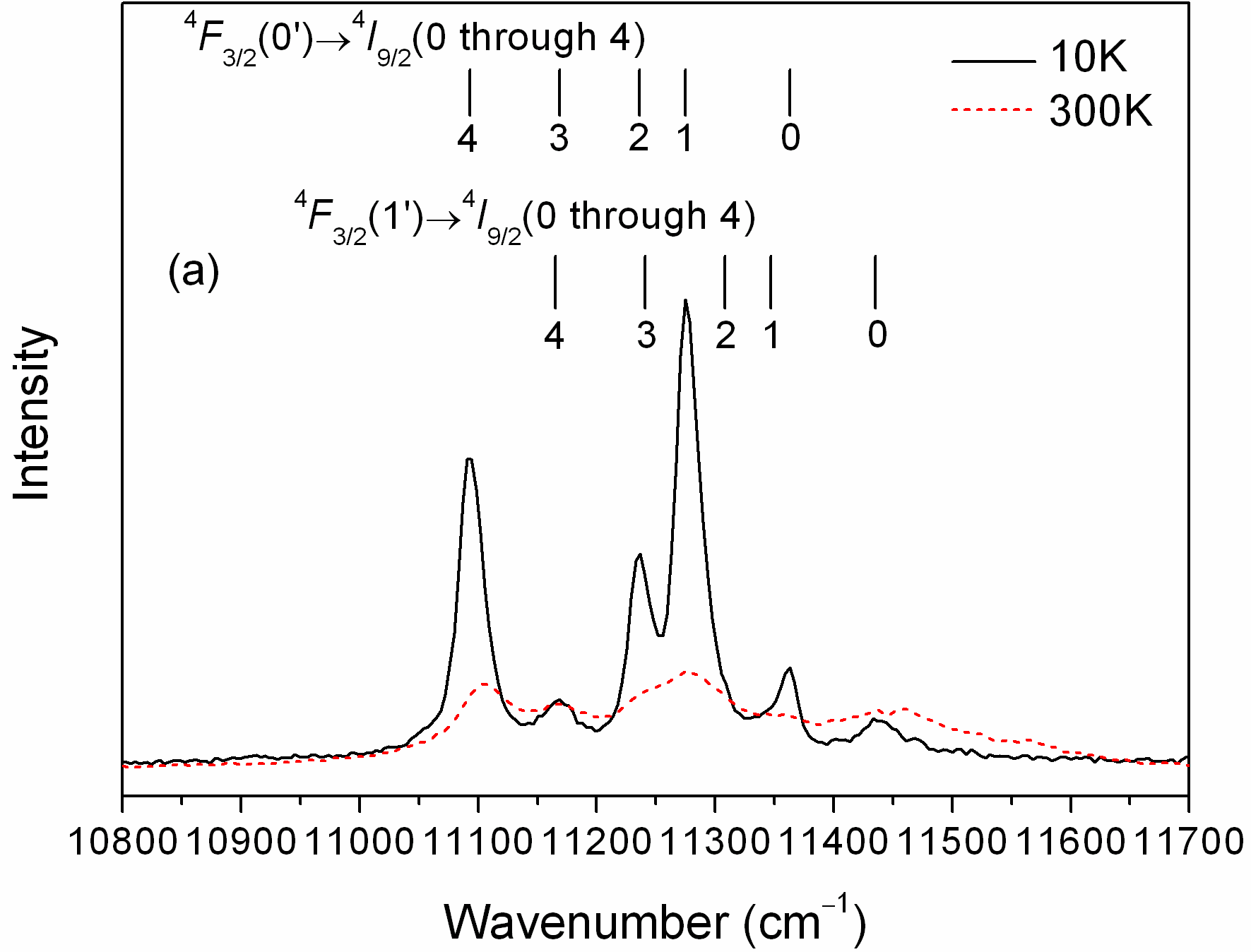


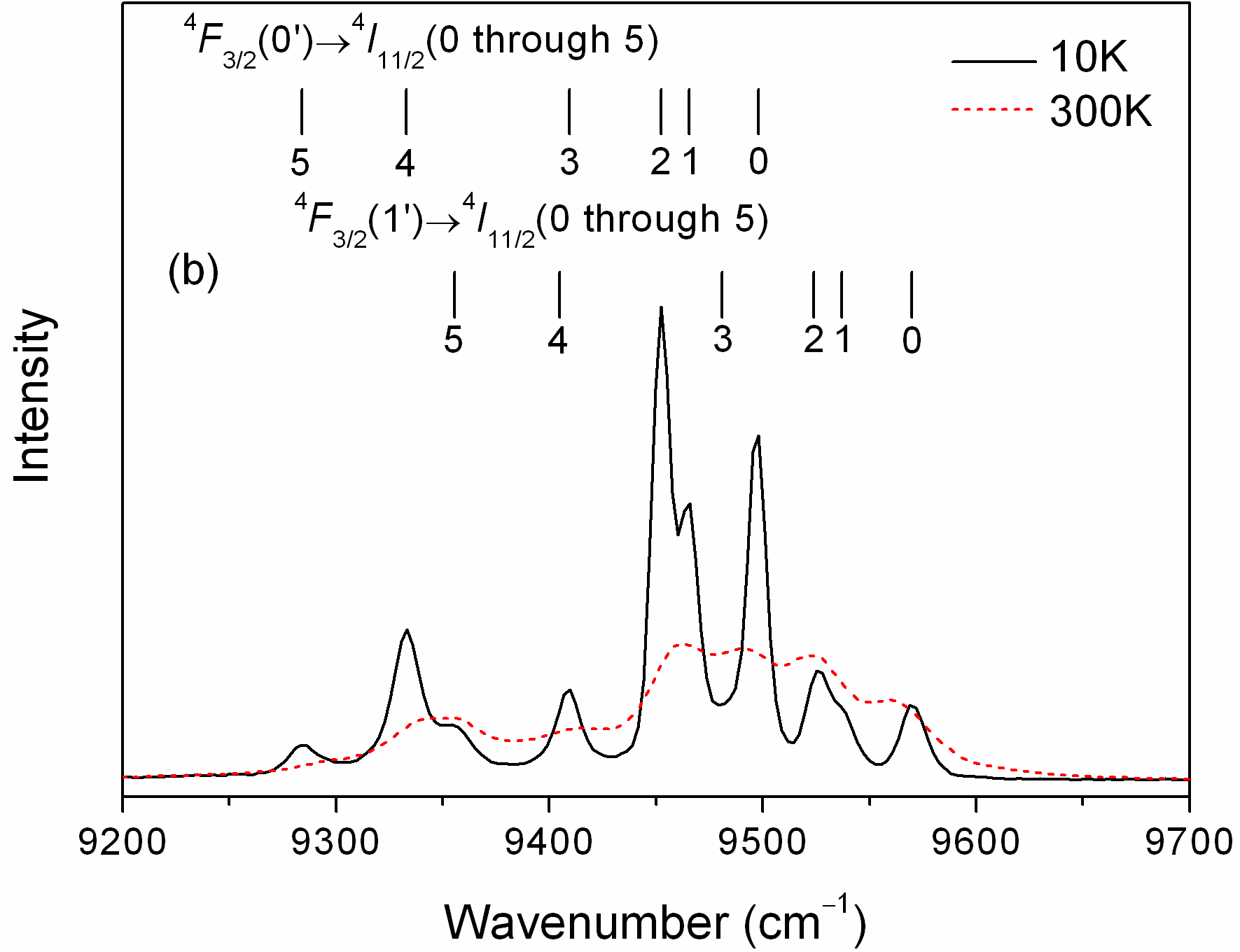


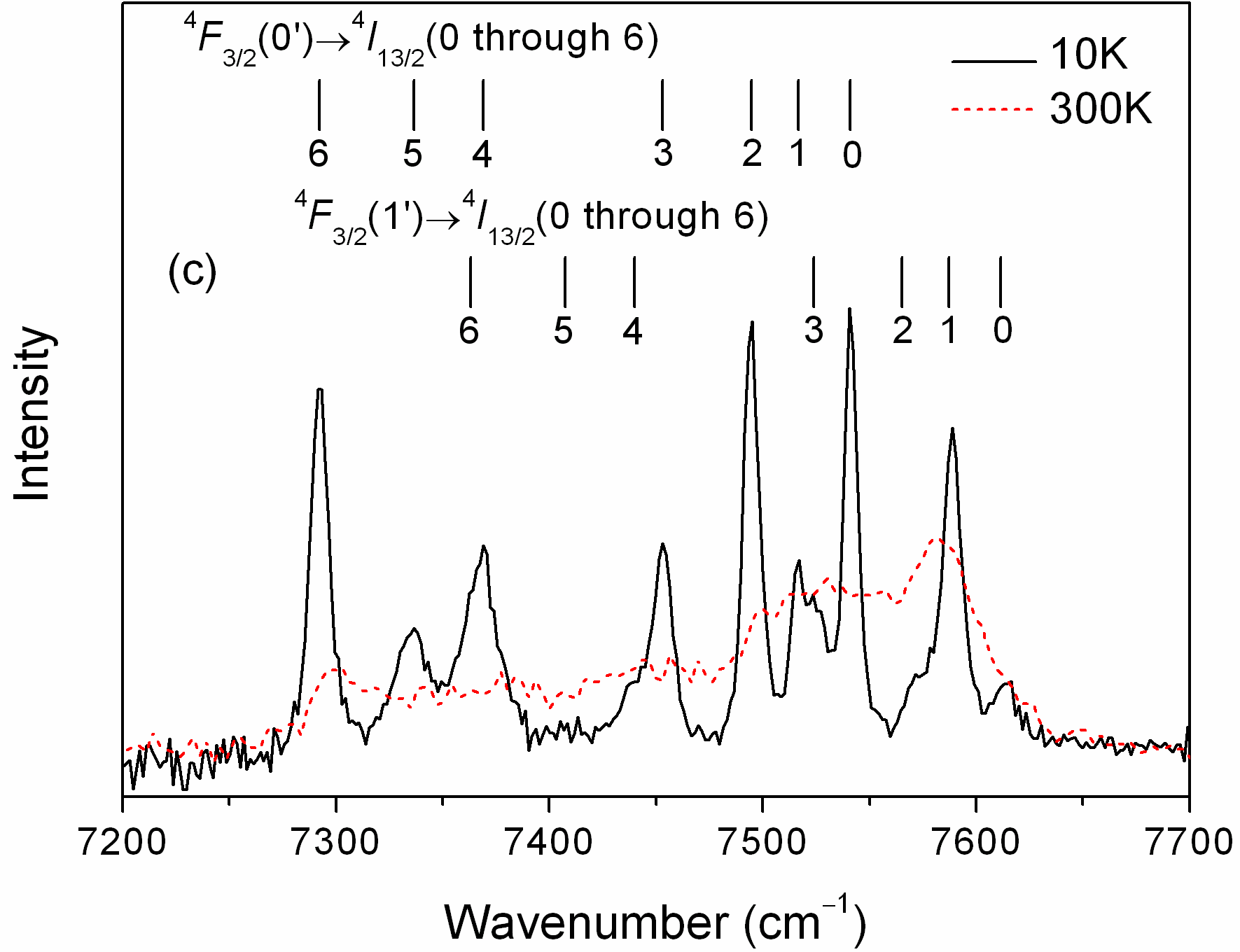

Supplement: Figure S5 — Emission fluorescence spectra of the Nd:KGdP4O12 crystal at 10K and 300K. (DOCX) [file pone.0100922.s005.docx]

**Figure S6.** Fluorescence decay curve of the 4*F*3/2 manifold of the Nd:KGdP4O12 crystal at room temperature.


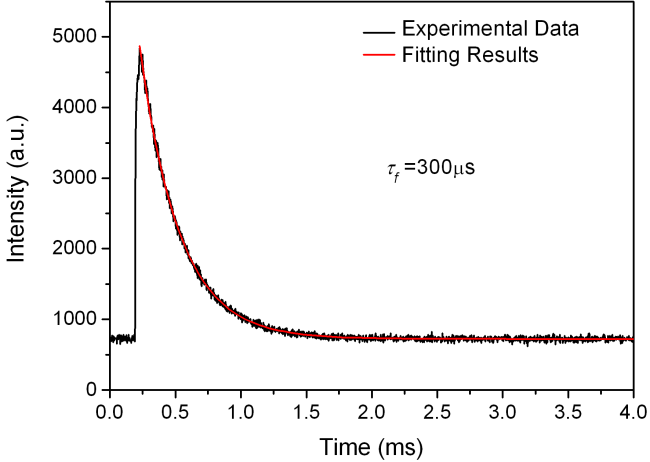

Supplement: Figure S6 — Fluorescence decay curve of the 4 F 3/2 manifold of the Nd:KGdP4O12 crystal at room temperature. (DOCX) [file pone.0100922.s006.docx]
