# Supplementary material for: Growth, Structure, Thermal Properties and Spectroscopic Characteristics of Nd3+-Doped KGdP4O12 Crystal
Source: PLoS One. 2014 Jun 26;9(6):e100922. doi: 10.1371/journal.pone.0100922 (PMC4072700; doi:10.1371/journal.pone.0100922)
Supplement: Table S1 — Crystal data, data collection and refinement of Nd:KGdP4O12. (DOCX) [file pone.0100922.s007.docx]

**Table S1.** Crystal data, data collection and refinement of Nd:KGdP_4_O_12_.

| **Crystal data** | **Data collection** | **Refinement** |
| --- | --- | --- |
| KGd_0.95_Nd_0.05_P_4_O_12_  formula weight = 511.58  monoclinic, *C*2/*c*  *a* = 7.812(2) Å  *b* = 12.307(3) Å  *c* = 10.474(2) Å  *β* = 110.84(3)°  *V* = 941.1(4) Å^3^  *Z* = 4  *D_x_* = 3.611 g/cm^3^  *D_m_* = 3.54 g/cm^3^  Mo *K*α radiation  λ = 0.71073 Å  cell parameters from 1720 reflections  *θ* = 2.0−27.9°  *μ* = 8.237 mm^−1^  *T* = 293 (2) K  block, pink  0.18 × 0.16 × 0.12 mm | Rigaku Saturn CCD area-detector diffractometer  *ω* and *ϕ* scans  absorption correction: multi-scan  2628 measured reflections  806 independent reflections  794 reflections with *I* > 2*σ*(*I*)  *R*_int_ = 0.0823  *θ*_max_ = 24.97°  *h* = −9 → 7  *k* = −14 → 14  *l* = −12 → 12 | Refinement on *F*^2^  R[*F*^2^ > 2*σ*(*F*^2^)] = 0.0383  *w*R(*F*^2^) = 0.0970  *S* = 1.159  806 reflections  84 parameters  *w* = 1/[*σ*^2^(*F*_o_^2^) + (0.0650*P*)^2^+ 1.7546*P*] where *P* = (*F*_o_^2^ + 2*F*_c_^2^)/3  (Δ/*σ*)_max_ = 0.001  Δ*ρ*_max_ = 2.313 e Å^−3^  Δ*ρ*_min_ = −2.021 e Å^−3^  extinction correction: SHELXL97  extinction coefficient: 0.0263(8)  scattering factors from *International Tables for Crystallography*, Vol. C |
