# Supplementary material for: Growth, Structure, Thermal Properties and Spectroscopic Characteristics of Nd3+-Doped KGdP4O12 Crystal
Source: PLoS One. 2014 Jun 26;9(6):e100922. doi: 10.1371/journal.pone.0100922 (PMC4072700; doi:10.1371/journal.pone.0100922)
Supplement: Table S2 — Atomic coordinates and equivalent isotropic displacement parameters of Nd:KGdP4O12. (DOCX) [file pone.0100922.s008.docx]

**Table S2.** Atomic coordinates and equivalent isotropic displacement parameters of Nd:KGdP_4_O_12_

| Atom | Wyckoff position | *x* | *y* | *z* | *U*(eq) | Occupancy |
| --- | --- | --- | --- | --- | --- | --- |
| K | 4*e* | 0.5 | 0.31663(12) | 0.25 | 0.0213(3) | 1 |
| Gd | 4*e* | 0 | 0.11945(2) | 0.25 | 0.00600(9) | 0.95 |
| Nd | 4*e* | 0 | 0.11945(2) | 0.25 | 0.00600(9) | 0.05 |
| P1 | 8*f* | -0.04155(12) | 0.32795(9) | 0.00079(9) | 0.0074(2) | 1 |
| P2 | 8*f* | 0.21677(12) | 0.47741(9) | -0.06092(10) | 0.0068(2) | 1 |
| O1 | 8*f* | 0.0938(4) | 0.3719(2) | -0.0719(3) | 0.0098(8) | 1 |
| O2 | 8*f* | 0.0595(3) | 0.2430(2) | 0.0977(3) | 0.0097(7) | 1 |
| O3 | 8*f* | -0.2112(3) | 0.2988(3) | -0.1135(3) | 0.0088(7) | 1 |
| O4 | 8*f* | -0.0690(4) | 0.4273(3) | 0.0879(3) | 0.0087(7) | 1 |
| O5 | 8*f* | 0.3514(4) | 0.4872(3) | 0.0814(3) | 0.0093(7) | 1 |
| O6 | 8*f* | 0.2793(4) | 0.4749(3) | -0.1783(3) | 0.0094(7) | 1 |
