# Supplementary material for: Growth, Structure, Thermal Properties and Spectroscopic Characteristics of Nd3+-Doped KGdP4O12 Crystal
Source: PLoS One. 2014 Jun 26;9(6):e100922. doi: 10.1371/journal.pone.0100922 (PMC4072700; doi:10.1371/journal.pone.0100922)
Supplement: Table S3 — Anisotropic displacement parameters (Å2) of Nd:KGdP4O12. (DOCX) [file pone.0100922.s009.docx]

**Table S3.** Anisotropic displacement parameters (Å^2^) of Nd:KGdP_4_O_12_ *^a^*

| Atom | *U*_11_ | *U*_22_ | *U*_33_ | *U*_23_ | *U*_13_ | *U*_12_ |
| --- | --- | --- | --- | --- | --- | --- |
| K | 0.0460(7) | 0.0047(7) | 0.0140(5) | 0 | 0.0115(5) | 0 |
| Gd | 0.00826(14) | 0.00254(19) | 0.00651(14) | 0 | 0.00179(11) | 0 |
| Nd | 0.00826(14) | 0.00254(19) | 0.00651(14) | 0 | 0.00179(11) | 0 |
| P1 | 0.0094(4) | 0.0042(5) | 0.0083(4) | 0.0001(4) | 0.0027(3) | 0.0007(4) |
| P2 | 0.0092(4) | 0.0028(5) | 0.0088(4) | -0.0007(4) | 0.0034(4) | -0.0010(4) |
| O1 | 0.0114(14) | 0.0058(17) | 0.0133(15) | 0.0006(10) | 0.0060(12) | -0.0020(10) |
| O2 | 0.0130(11) | 0.0038(15) | 0.0117(11) | 0.0025(11) | 0.0038(10) | -0.0013(12) |
| O3 | 0.0108(11) | 0.0023(16) | 0.0130(11) | -0.0014(11) | 0.0040(10) | -0.0026(11) |
| O4 | 0.0094(11) | 0.0074(17) | 0.0076(11) | -0.0014(12) | 0.0009(10) | -0.0019(13) |
| O5 | 0.0126(11) | 0.0046(15) | 0.0105(12) | -0.0024(11) | 0.0039(10) | -0.0011(12) |
| O6 | 0.0142(12) | 0.0053(16) | 0.0089(13) | 0.0008(11) | 0.0042(11) | 0.0011(12) |

*^a^* The anisotropic displacement factor exponent takes the form −2π^2^Σ*_i_*Σ*_j_U_ij_h_i_h_j_a_i_*^*^*a_j_*^*^.
