# Supplementary material for: Growth, Structure, Thermal Properties and Spectroscopic Characteristics of Nd3+-Doped KGdP4O12 Crystal
Source: PLoS One. 2014 Jun 26;9(6):e100922. doi: 10.1371/journal.pone.0100922 (PMC4072700; doi:10.1371/journal.pone.0100922)
Supplement: Table S4 — Bond lengths and bond valences in the Nd:KGdP4O12 crystal. (DOCX) [file pone.0100922.s010.docx]

**Table S4.** Bond lengths and bond valences in the Nd:KGdP_4_O_12_ crystal.

| Atom A | Atom B | Bond length (Å) | Bond valence *s*_ij_ | Σ*s*_ij_ |
| --- | --- | --- | --- | --- |
| Gd(Nd) | O2 × 2 | 2.3665 × 2 | 0.445 × 2 | 3.319 |
| Gd-Occ=0.95 | O5 × 2 | 2.3783 × 2 | 0.431 × 2 |  |
| Nd-Occ=0.05 | O6 × 2 | 2.4095 × 2 | 0.396 × 2 |  |
|  | O3 × 2 | 2.4189 × 2 | 0.386 × 2 |  |
| K | O5 × 2 | 2.7229 × 2 | 0.202 × 2 | 1.051 |
|  | O1 × 2 | 2.903 × 2 | 0.124 × 2 |  |
|  | O3 × 2 | 2.909 × 2 | 0.122 × 2 |  |
|  | O6 × 2 | 3.3227 × 2 | 0.040 × 2 |  |
|  | O2 × 2 | 3.3617 × 2 | 0.036 × 2 |  |
| P1 | O2 | 1.4752 | 1.467 | 5.051 |
|  | O3 | 1.4796 | 1.450 |  |
|  | O4 | 1.5851 | 1.090 |  |
|  | O1 | 1.6009 | 1.044 |  |
| P2 | O6 | 1.4759 | 1.464 | 4.977 |
|  | O5 | 1.4924 | 1.400 |  |
|  | O1 | 1.5948 | 1.062 |  |
|  | O4 | 1.5986 | 1.051 |  |
| O1 | P2 | 1.5948 | 1.062 | 2.231 |
|  | P1 | 1.6009 | 1.044 |  |
|  | K1 | 2.9030 | 0.124 |  |
| O2 | P1 | 1.4752 | 1.467 | 1.948 |
|  | Gd(Nd) | 2.3665 | 0.445 |  |
|  | K | 3.3617 | 0.036 |  |
| O3 | P1 | 1.4796 | 1.450 | 1.959 |
|  | Gd(Nd) | 2.4189 | 0.386 |  |
|  | K | 2.9090 | 0.122 |  |
| O4 | P1 | 1.5851 | 1.090 | 2.141 |
|  | P2 | 1.5986 | 1.051 |  |
| O5 | P2 | 1.4924 | 1.400 | 2.034 |
|  | Gd(Nd) | 2.3783 | 0.431 |  |
|  | K | 2.7229 | 0.202 |  |
| O6 | P2 | 1.4759 | 1.464 | 1.901 |
|  | Gd(Nd) | 2.4095 | 0.396 |  |
|  | K | 3.3227 | 0.040 |  |
