# Supplementary material for: Growth, Structure, Thermal Properties and Spectroscopic Characteristics of Nd3+-Doped KGdP4O12 Crystal
Source: PLoS One. 2014 Jun 26;9(6):e100922. doi: 10.1371/journal.pone.0100922 (PMC4072700; doi:10.1371/journal.pone.0100922)
Supplement: Table S5 — Frequencies (cm−1) and assignments of IR absorption and Raman scattering for Nd:KGdP4O12. (DOCX) [file pone.0100922.s011.docx]

**Table S5.** Frequencies (cm^−1^) and assignments of IR absorption and Raman scattering for Nd:KGdP_4_O_12_.

| IR | Raman | Assignment |
| --- | --- | --- |
| 1286 s 1249 s | 1265 w 1244 m 1220 w | *ν*_as_(O−P−O) |
| 1127 s  1117 sh | 1179 s | *ν*_s_(O−P−O) |
| 1031 vs 1006 s | 1122 vw 1081 w | *ν*_as_(P−O−P) |
| 736 m 712 m  700 sh | 797 vw 684 vs | *ν*_s_(P−O−P) |

vs−very strong, s−strong, m−medium, w−weak, vw−very weak, sh−shoulder
